# Cross-Sensor Fingerprint Enhancement Using Adversarial Learning and Edge Loss

**DOI:** 10.3390/s22186973

**Published:** 2022-09-15

**Authors:** Ashwaq Alotaibi, Muhammad Hussain, Hatim AboAlSamh, Wadood Abdul, George Bebis

**Affiliations:** 1 Department of Computer Science, CCIS, King Saud University, Riyadh 11451, Saudi Arabia; 2 Department of Computer Engineering, King Saud University, Riyadh 11451, Saudi Arabia; 3 Department of Computer Science and Engineering, University of Nevada, Reno, NV 89557, USA

**Keywords:** biometrics, cross-sensor fingerprints, fingerprint enhancement, cGAN, adversarial learning, deep learning

## Abstract

A fingerprint sensor interoperability problem, or a cross-sensor matching problem, occurs when one type of sensor is used for enrolment and a different type for matching. Fingerprints captured for the same person using various sensor technologies have various types of noises and artifacts. This problem motivated us to develop an algorithm that can enhance fingerprints captured using different types of sensors and touch technologies. Inspired by the success of deep learning in various computer vision tasks, we formulate this problem as an image-to-image transformation designed using a deep encoder–decoder model. It is trained using two learning frameworks, i.e., conventional learning and adversarial learning based on a conditional Generative Adversarial Network (cGAN) framework. Since different types of edges form the ridge patterns in fingerprints, we employed edge loss to train the model for effective fingerprint enhancement. The designed method was evaluated on fingerprints from two benchmark cross-sensor fingerprint datasets, i.e., MOLF and FingerPass. To assess the quality of enhanced fingerprints, we employed two standard metrics commonly used: NBIS Fingerprint Image Quality (NFIQ) and Structural Similarity Index Metric (SSIM). In addition, we proposed a metric named Fingerprint Quality Enhancement Index (FQEI) for comprehensive evaluation of fingerprint enhancement algorithms. Effective fingerprint quality enhancement results were achieved regardless of the sensor type used, where this issue was not investigated in the related literature before. The results indicate that the proposed method outperforms the state-of-the-art methods.

## 1. Introduction

The fingerprint is a biometric modality deployed mainly for human identification. Fingerprint recognition systems have several practical applications, including access control and criminal investigation [1].

Most available fingerprint systems compare data captured from the same sensor, where matching algorithms are designed to work on data obtained from a single sensor for enrollment and verification. Thus, the ability of these algorithms to work on data collected from multiple sensors is limited. It is known as the fingerprint sensor interoperability problem or the cross-sensor problem. In legacy databases, billions of fingerprints have been collected from different sensors based on diverse technologies. Every time the sensor of choice is changed, the re-enrollment of persons is a costly and substantial task. Moreover, due to the improvement in fingerprint sensors and the need to apply fingerprint recognition in devices such as those linked to the Internet of Things (IoT), the demand is high for an efficient fingerprint matching algorithm that can recognize fingerprints captured using different sensors. Therefore, the algorithms for the sensor interoperability problem, which improve the biometric system’s ability to adapt to data obtained from several sensors, are highly needed and will significantly impact system usability [2].

**Figure 1:**
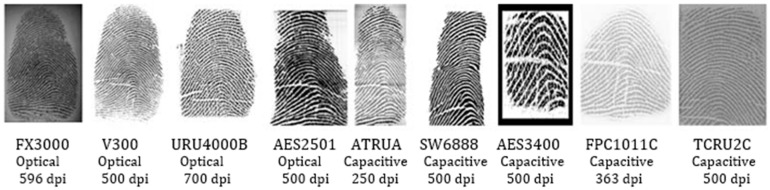
Fingerprints from the FingerPass database of the same finger that were captured by different sensors.

The quality of fingerprints varies based on the sensor types used for capturing the fingerprint, even if the same sensing technology is employed (e.g., optical or capacitive). Additionally, the corresponding sets of features have high variability, which cannot be analyzed easily by a matching algorithm for accurate decisions. An example is shown in Figure 1, which shows the fingerprint of the same finger captured by nine different sensors [3].

Differences in sensor technology and interaction type can cause significant variations in the quality of fingerprints. Thus, a considerable drop in the performance of the existing fingerprint recognition systems has been reported when different sensors are used for identification [2].

Moreover, the performance of cross-sensor matching algorithms is affected because of the variations in ridge patterns caused by the various types of noises and artifacts due to the difference in sensor technologies, as shown in Figure 1. There is a real need to enhance fingerprint images. However, this is challenging because fingerprints captured using various sensors include several kinds of texture patterns and noises [4].

**Figure 2:**
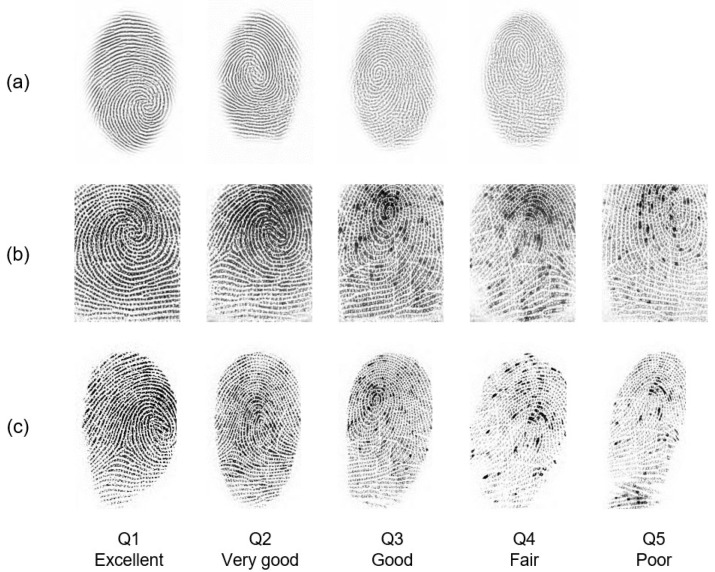
Quality variations *Q* = {1, 2, 3, 4,5} per impression for the same subject across three sensors: (**a**) Lumidigm Venus, (**b**) Secugen Hamster-IV, and (**c**) CrossMatch L-Scan Patrol from MOLF dataset.

A sample including a set of impressions taken from the MOLF dataset [5] is presented in Figure 2. These impressions were categorized into three subsets: DB1 comprises the flat dap (10) fingerprints captured by the Lumidigm Venus sensor; DB2 contains the fingerprints of the same fingers captured by the Secugen HamsterIV sensor; and DB3 consists of the dap fingerprints captured by CrossMatch L-Scan patrol sensor. Their quality was measured using the NFIQ (NBIS Fingerprint Image Quality) tool [6]. It is an open-source minutiae-based quality evaluation algorithm that provides a quality value {1, 2, 3, 4, 5}, with 1 representing the best quality and 5 denoting the worst one. Each row within the set stands for fingerprints captured by the same sensor. Each column, in turn, represents the same level of quality, in which the first column is excellent while the last column is poor. It can be noticed that DB1 has no images of the poor class. In addition, most of the ridge pattern information is unclear in the impressions belonging to classes poor and fair in DB2 and DB3.

In this paper, we present an efficient enhancement solution for the cross-sensor fingerprint problem. Specifically, motivated by the outstanding performance of deep learning-based techniques in various computer vision tasks such as image enhancement [7,8]. We designed an image-to-image mapping function ℱ that receives a low-quality fingerprint and generates a high-quality one. We model ℱ using Convolutional Neural Networks (CNN) based on encoder–decoder architecture. The learning of this kind of CNN is a challenging problem. Thus, we trained our method using two types of learning approaches i.e., the conventional end-to-end approach and using the adversarial learning (using a conditional GAN framework).

Adversarial learning generates fingerprints of higher quality than those produced by conventional learning, as demonstrated by comparing the outputs of the two methods using two frequent metrics: NFIQ and SSIM.

Our method was evaluated on two benchmark public datasets, FingerPass and MOLF. The results indicate that fingerprints are enhanced to higher quality regardless of the sensor type used.

To the best of our knowledge, this is the first work dealing with the problem of cross-sensor fingerprint enhancement using deep learning. Our contributions in this paper can be summarized as follow:

- We formulated the cross-sensor fingerprint enhancement problem as an image-to-image transformation problem and designed it using a CNN model with an encoder–decoder architecture that takes a low-quality fingerprint and produces an enhanced fingerprint. We trained the proposed CNN model using two different approaches: conventional learning and adversarial learning.
- Motivated by the success of adversarial learning in modeling image-to-image transformation [9], we learned the proposed image-to-image transformation (the CNN model) using a conditional GAN framework, where the proposed CNN model plays the role of a generator.
- To preserve the ridge patterns in the fingerprints, we incorporated the edge loss function [10] and *L*1 loss [9] into the adversarial loss [11]. This resulted in good quality enhanced fingerprints regardless of the type of sensor used to capture the fingerprints.
- For comprehensive evaluation of a fingerprint enhancement algorithm, we proposed a new metric called Fingerprint Quality Enhancement Index (FQEI). This metric yields a value between 1 and −1, where 1 represents the best enhancement and −1 represents the worst degradation.

The rest of this paper is structured as follows. Section 2 reviews previous enhancement methods, while Section 3 describes in detail the proposed method. Section 4 presents the training and testing stages of our model, while Section 5 gives details of the experiments. Section 6 discusses our results. Finally, Section 7 concludes the conducted work and suggests some directions for future work.

## 2. Related Work

In the last decade, various studies have been conducted to study the effect of reliable fingerprint enhancement for solving the matching problem assuming that the same sensor was used both for enrollment and verification.

A common technique is the HONG method proposed by Hong et al. [12], where fingerprints are enhanced using a bank of Gabor filters, which are adjusted to the orientation of the local ridges. Another state-of-the-art method is the CHIK method, which was proposed by Chikkerur et al. [13], where fingerprints are enhanced using the short-time Fourier transform (STFT). In this method, each fingerprint is initially divided into small overlapping windows, and the STFT is applied to each window. Next, the block energy, ridge orientation, and ridge frequency are estimated using the Fourier spectrum, and then contextual filtering is applied for fingerprint enhancement.

Other enhancement techniques focus on using off-line images, such as the latent fingerprint technique [14]. Researchers proposed an approach that employed a CNN model to predict ridge direction from a set of pre-trained ridge patterns. In [7], a direct end-to-end enhancement approach was proposed using the FingerNet architecture. This method relied on the use of a CNN within an encoder–decoder scheme. In [8], the authors employed a convolutional auto-encoder neural network to enhance the missing ridge pattern. A similar work was proposed in [15], where a method based on de-convolutional auto-encoders was developed to match sensor-scan and inked fingerprints.

All previous works have focused on using conventional learning only in the enhancement process, where CNNs learn to minimize the loss function. This process, however, requires a lot of manual effort. In contrast, the flexibility provided by Generative Adversarial Networks (GANs), which apply adversarial learning, allows for optimizing the objective function of the problem more effectively. It initially determines a single high-level goal, such as producing indistinguishable fake images from real images, and then learns to achieve such a goal automatically using a suitable loss function [9]. In the JOSHI method [16], a conditional GAN model was proposed based on an image-to-image translation to reconstruct the ridge structure of latent fingerprints. As discussed above, most previous enhancement methods have focused on matching latent fingerprints left unintentionally at a crime scene. Unlike previous methods, which deal with latent fingerprints, the proposed method addresses the problem of enhancing cross-sensor fingerprints. The problem of cross-sensor enhancement has been addressed in a few studies only. In [4,17], an adaptive histogram equalization method was proposed to enhance the contrast of contactless fingerprint ridges and valleys. To date, these are the only published studies concerning cross-sensor enhancement. No previous studies have addressed the cross-sensor enhancement problem using deep learning techniques.

## 3. Proposed Method

A critical issue when designing an effective cross-sensor fingerprint enhancement is preserving valleys, ridges, and other fingerprint features, such as minutiae. In view of this, we introduce a new method for cross-sensor fingerprint enhancement.

### 3.1. Problem Formulation

Fingerprint enhancement can be expressed as an image-to-image transformation problem. It aims to learn a mapping, denoted by ℱ, which transforms an input fingerprint $x\in {\mathbb{R}}^{mxn}$ to an enhanced fingerprint $\hat{y}$. This implies finding a mapping $ℱ:{\mathbb{R}}^{mxn}\to {\mathbb{R}}^{mxn}$ such that $\hat{y}=ℱ\left(x;\;\theta \right)$, where θ represents the transformation parameters. A critical question in this context is how to model the mapping function  ℱ. From a practical standpoint, the application of both deep learning and CNNs has shown promising performance in pattern recognition problems, as indicated in various studies [4,14,15]. This, in turn, motivated us to model ℱ using a CNN model. The learning method typically employed in CNNs is conventional learning, which is based on an objective function that minimizes the loss function between ground truth and the predicted labels. However, regardless of whether the learning process is automatic, several studies have sought to design more effective loss functions [9].

Another efficient learning approach is based on the Generative Adversarial Networks (GANs) framework. The learning method applied in GANs is adversarial learning, which is based on a min-max game and includes a specific loss function, where one agent tries to maximize while the other one tries to minimize [11]. 

### 3.2. The Design of Mapping Function (ℱ)

The design of the mapping function (ℱ) is a challenging problem since the captured fingerprints by different sensors have different texture patterns and noise [4]. The desired mapping must be developed to enhance fingerprints by preserving the underlying fingerprint features and removing possible corruption and noise. To address these issues and effectively learn ℱ, two learning frameworks were investigated: conventional learning and adversarial learning.

#### 3.2.1. Conventional Learning Framework (One-Net)

In this case, ℱ was designed using a CNN model following an encoder–decoder architecture [18]. It takes a low-quality fingerprint as input and produces a high-quality one as output. This architecture minimizes the loss between the target images and the predicted ones. This architecture was adopted from SegNet [19] with some modifications. SegNet comprises two networks: an encoder and a corresponding decoder, followed by a final pixel-wise classification layer.

SegNet has five encoders and corresponding five decoders. All the encoders include two consecutive layers and max pooling layers. Each convolutional layer consists of 64 filters with size 3 × 3, 1 padding and stride of 1 followed by batch normalization (BN) layer and then element-wise rectified linear non-linearity (ReLU). After that, 2 × 2 max pooling layer, with a stride of 2, is applied where the related max pooling indices (locations) are saved.

Each corresponding decoder up-samples its input using the recalled max-pooling indices using a 2 × 2 max unpooling layer with a stride of 2. Then, it convolves the input using two consecutive convolutional layers. Each convolutional layer contains 64 filters of size 3 × 3 and a stride of 1 followed by a batch normalization layer, then a ReLU layer. The final output is then fed into a multi-class soft-max classifier to compute class probabilities for each pixel independently.

**Figure 3:**
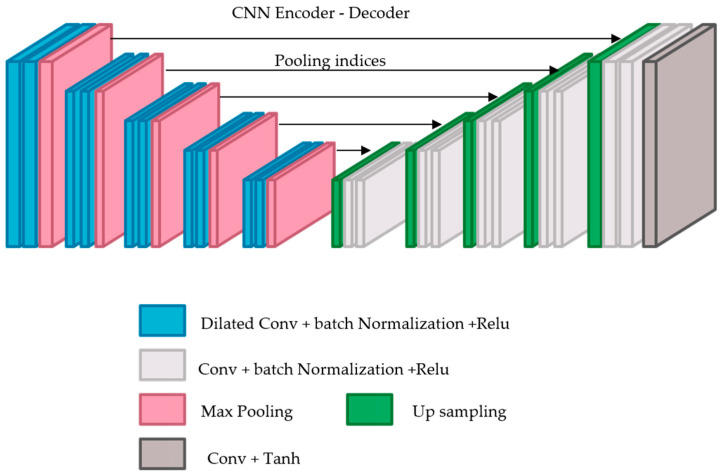
Conventional learning framework.

This model has been specifically designed for segmentation purposes. However, since our goal is different and focuses on the enhancement task, the SegNet model was modified to fit the task of interest by receiving a low-quality, 300 × 300 × 1 fingerprint and generating a same-size fingerprint with higher quality. Both the Softmax layer and the pixel-wise classification layer were removed. Since the target task is to produce a same-size fingerprint with a higher quality, a convolution layer with one filter of size 3 × 3, was also added, as shown in Figure 3.

**Figure 4:**
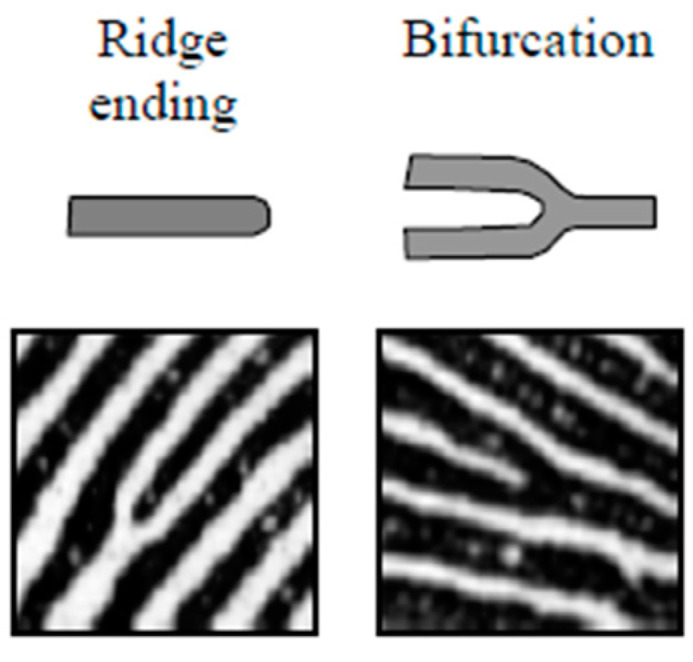
The two most common minutiae—ridge ending and bifurcation. Reprinted with permission from Ref. [1]. Copyright 2022, Springer Nature.

The preservation of small and thin details is essential for fingerprint matching since they play an important role in determining the identity of each subject. Some of these details are the minutiae points formed mainly by ridge bifurcations and ridge endings. The ridge bifurcations are those points where ridges are divided into two ridges, whereas the ridge endings are those points where ridges end. The extraction of minutiae points is a difficult task in low-quality fingerprint images [1], see Figure 4.

These small details should be considered when designing the target model. Convolutional networks are deployed to gradually reduce the image resolution until it is represented via tiny feature maps, where the spatial structure is not yet visible. However, this spatial acuity loss may restrict fingerprint enhancement. This issue can be addressed by dilated convolutions that can increase the output feature maps resolution without decreasing the individual neurons’ receptive field. Thus, a second modification introduced to the SegNet model is adding dilated convolutions.

Generally, dilated convolution is a convolution having a wider kernel that is generated based on repeatedly adding spaces among the kernel elements [20]. Therefore, each convolution layer in the encoder was substituted by a dilated convolution layer using a different dilation factor in the range: 1, 1, 2, 2, 4, 4, 8, 8, 16, and 16. Our results illustrate that dilated convolution is appropriate for fingerprint enhancement since it enlarges the receptive field with no coverage or resolution loss.

In the decoder network, each decoder up-samples its input feature map(s) by deploying the memorized max-pooling indices related to its corresponding encoder’s feature map(s). It should be noted that there is no conducted learning within the up-sampling stage. SegNet uses the max pooling indices to up-sample the feature map(s) and convolves them with a trainable decoder filter bank. Next, batch normalization is applied to each map. Subsequently, the high dimensional feature representation at the final decoder output is fed to a convolutional layer followed by a Tanh layer as shown in Table 1.

#### 3.2.2. The Adversarial Learning Framework (Two-Net)

This type of learning is based on the conditional generative adversarial network (cGAN) framework [9]. The cGAN framework consists of a generator and a discriminator. The role of the generator is to produce a transformed image from the input one. The discriminator determines if the input image is real or fake. In the training stage, both the generator and discriminator conduct a min-max game. For this task, ℱ plays the role of the generator, which is to produce a high-quality fingerprint ($\hat{y}$) from a low-quality one (x). The enhanced high-quality fingerprint must have a clear ridge structure to preserve the valleys, ridges, and further fingerprint features, such as minutiae points. The discriminator differentiates real fingerprints from the generated ones, which helps to learn ℱ.

**Figure 5:**
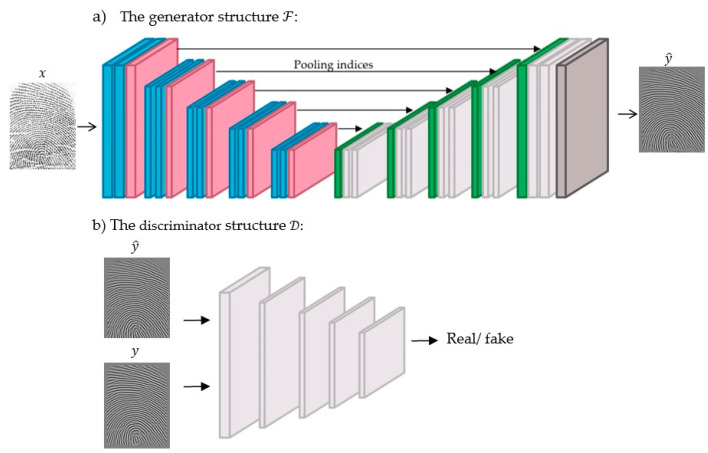
The adversarial learning framework. The blue color represents dilated Conv, BN and ReLU layers; the pink color represents Max-Pooling layer; the green color represents up sampling layer; light grey color represents Conv, BN and ReLU layers; the dark grey color represents Conv and Tanh layers.

To effectively learn ℱ via the cGANs framework, it is considered a generator that generates an enhanced image $\hat{y}$ from an input image x. To model ℱ, a dilated SegNet is deployed since both the input and output are images with the same size 300 × 300 × 1, as explained in the first framework. The discriminator D is modeled using a patch GAN discriminator that was adopted from the paper [9]. The first convolution layer Conv contains 64 filters, stride 2, depth of 2, followed by a Leaky ReLU layer. The second Conv consists of 128 filters, stride 2, and the third contains 256 filters of stride 2; the fourth Conv contains 512 filters, stride 2; each of these layers is followed by a batch normalization layer and the Leaky ReLU. The last layer is a Conv layer consisting of one filter and stride of 1. All these Conv layers contain filters of size 4, as illustrated in Figure 5.

### 3.3. Loss Functions and the Learning of (ℱ)

For the first framework, ℱ is learned through conventional learning based on taking a low-quality fingerprint x and producing a high-quality one. This model minimizes the gradient difference between the generated fingerprint and the ground truth y. We used two loss functions: *L*1 loss [9] and Edge Loss [10].

The first loss used is the L1 distance that is expressed as follows:

(1) $${ℒ}_{L1}\left(ℱ\right)=E_{x,y}\left[∥y-ℱ{(x)∥}_{1}\right]$$

An ideal fingerprint image has valleys and ridges that flow in a locally regular direction. In this case, the detection of ridges is straightforward, and minutiae can be accurately located within the image. Nevertheless, skin conditions (e.g., dry/wet, bruises, and cuts), improper finger pressure, and sensor noise significantly impact fingerprint image quality.

Therefore, the edge loss function is added to improve the fingerprint ridge structures by calculating the edge direction. For this, the ridge pattern of the generated fingerprint and the corresponding ground truth fingerprint are initially computed, and then the loss is used to update the parameters of ℱ. The edge loss is denoted as $L_{edge}$ and can be expressed as follows:

(2) $${ℒ}_{edge}\left(ℱ\right)=\sqrt{∥∆ℱ\left(x\right)-∆{y∥}^{2}+\varepsilon^{2}}$$

where ∆ represents the Laplacian of Gaussian operator, y denotes the ground truth fingerprint (high quality), and ℱ(x) denotes the enhanced image. The parameter with constant ε empirically set to $10^{-3}$ as used in [10]. This loss is used to preserve edge features useful for improving ridge patterns.

The total loss

(3) $${ℒ}_{Conventional}\left(ℱ\right)=\mu \;{ℒ}_{L1}\left(ℱ\right)+\lambda {ℒ}_{edge}\left(ℱ\right).$$

In the second framework, ℱ learning is inspired by the method [9]. Both D and ℱ are learned using adversarial learning. The training dataset includes pairs of poor- and high-quality fingerprints. Such pairs are expressed as $\left(x_{i};\;y_{i}\right)$, in which $x_{i}$ stands for the poor-quality fingerprint image, while $y_{i}$ stands for the corresponding high-quality one (ground truth).

A fingerprint x is fed into ℱ, which then maps it to an enhanced version $\hat{y}$. The channel-wise concatenation between the pairs (x, y) and $\left(x,\hat{y}\right)$ is then fed into D to classify them as real or generated fingerprints. The discriminator ensures that the generator effectively learns to preserve ridge structures of the generated enhanced fingerprints. The adversarial loss is given below:

(4) $${ℒ}_{GAN}\left(ℱ,\;D\right)=E_{\left(x,y\right)}\left[\log(D\left(x,y\right)+E_{x}[\log(1-D\left(x,ℱ\left(x\right)\right)\;\right]].\;$$

A custom training loop is deployed to train the model using the training dataset, in which the network weights are updated in each iteration. In the training stage, ℱ produces a fingerprint that is hard to be classified as synthetic via D. In contrast, D avoids being misled by ℱ and increases the successful discrimination between the original and synthetic fingerprints by reducing the value of the loss function.

We combined the edge loss and *L*1 distance with adversarial learning. The final objective function is expressed below:

(5) $$arg\min ℱ\max D\;{ℒ}_{GAN}(ℱ,\;D)+\mu \;{ℒ}_{L1}\left(ℱ\right)+\lambda {ℒ}_{edge}\left(ℱ\right).$$

**Figure 6:**
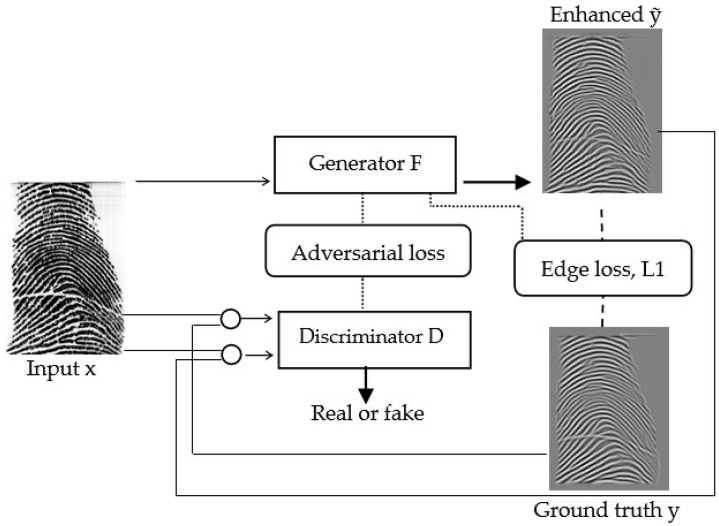
The learning procedure of ℱ using adversarial learning. The thin arrows represent the input; the thick arrows represent the output; The dotted lines represent weights updating, the dashes represent the two fingerprints used to calculate the edge loss and *L*1 loss; and the circles represent the channel-wise concatenation.

Figure 6 illustrates the training framework, which learns ℱ to produce an enhanced fingerprint from an input one.

### 3.4. Assessing the Quality of the Enhancements

Although both NFIQ [6] and SSIM [21] are popular and accurate metrics used widely to measure fingerprint quality, they do not offer a comprehensive description of what happens during enhancement. In these metrics, the number of enhanced or degraded images is not considered. A new metric has been designed to comprehensively describe each class’s performance by analyzing the NFIQ results.

#### Fingerprint Quality Enhancement Index (FQEI)

The detail of the new metric for assessing the enhancement potential of an algorithm is given in the following paragraphs. A fingerprint can be assigned to one of five quality levels, i.e., *Q*1: excellent, *Q*2: very good, *Q*3: good, *Q*4: fair, or *Q*5: poor, based on the scores obtained from the NFIQ tool [6]. Using the quality levels of fingerprints before and after enhancement, we compute the Quality Confusion Matrix (QCM) as shown in Table 2, where *Q_jj_* is the number of images with original quality Q*_j_* have been enhanced to quality $Q_{i}$.

To quantify the enhancement quality, each *Q*_jj_ in QCM is scaled according to the corresponding coefficient *w_ij_* in the weight quality matrix (WQM), shown in Table 3.

In WQM, (i) *w_ii_ =* 0 because there is no enhancement in the quality level of the fingerprints, (ii) *w_ij_* (*i < j*) is 1, 2, 3, or 4 depending on enhancement levels, e.g., in case of *Q*_13_, the quality of fingerprints after enhancement goes two levels up from *Q*_3_ to *Q*_1_, it must be weighted with *w*_13_ = 2, (iii) *w_ij_* (*i > j*) is −1, −2, −3 or −4 depending on de-enhancement levels.

The enhancement score ($E_{s}$), which quantifies the quality of enhancement of fingerprints that were in a low-quality class before enhancement and assigned to a high-quality class after enhancement, can be expressed using QCM and WQM as follows:

(6) $$E_{s}=\overset{5}{\underset{j=2}{\sum }}\overset{}{\underset{j>i}{\sum }}Q_{ij}\times w_{ij}$$

The degradation score ($D_{s}$), which quantifies the quality of de-enhancement of fingerprints that were in a high-quality class before enhancement and assigned to a low-quality class after enhancement, can be expressed using QCM and WQM as follows:

(7) $$D_{s}=\overset{5}{\underset{i=2}{\sum }}\overset{}{\underset{j<i}{\sum }}Q_{ij}\times w_{ij}$$

In the ideal case (IS) scenario, all images are enhanced from low-quality class to excellent class. In other words, IS can be represented as a weighted sum of all images, except those of *Q*1 quality, using the following formula:

(8) $$IS=\left(Q_{12}\times 1\right)+\left(Q_{13}\times 2\right)+\left(Q_{14}\times 3\right)+\left(Q_{15}\times 4\right)$$

where *Q*_12_ represents images from very good class that enhanced one degree up to be in class excellent, and so on.

However, in the worst-case (WS) scenario, all images move from the high-quality class to the poor-quality class, excluding the class poor since its images preserve their class. This means that WS can be expressed as a weighted sum of all images, except those in class poor, using the following formula:

(9) $$WS=\left(Q_{51}\times -4\right)+\left(Q_{52}\times -3\right)+\left(Q_{53}\times -2\right)+\left(Q_{54}\times -1\right)$$

where $Q_{51}$ represents images from excellent class that degraded four degrees down to be in poor class, and so on.

To measure the enhancement ratio (*ER*), the $E_{s}$ computed using Equation (6), is divided by *IS* computed using Equation (8). Thus, the *ER* is expressed as follows:

(10) $$ER=\frac{E_{s}\;}{IS}$$

In contrast, the degradation ratio can be measured by dividing the $D_{s}$ by *WS* as follows:

(11) $$DR=\frac{D_{s}}{WS}$$

The difference between the enhancement ratio and the degradation ratio is computed to determine the degree of enhancement for measuring the performance of an algorithm:

(12) FQEI =ER−DR.

In the ideal case scenario FQEI = 1, and it is equal to −1 in the worst-case scenario.

The more the FQEI is close to one, the higher the enhancement is, and vice versa. An illustrative example is provided in the Appendix A.

## 4. Training and Testing

In this section, we discuss the training stage, which uses training data to learn the model, and the testing stage tests it using test data.

### 4.1. Training Details

The model constructed is a supervised generative one trained to generate high-quality fingerprint images from low-quality ones. Practically, a supervised model needs paired training data of low-quality fingerprints combined with their corresponding enhanced images. However, cross-sensor fingerprint datasets have low-quality fingerprints, and their related high-quality counterparts are not available. Moreover, cross-sensor fingerprint databases are not large enough with high-quality images. This results in training difficulties of deep neural network models. Therefore, there is a need to generate fingerprints with noise characteristics similar to those of real fingerprints, as shown in Figure 1, and their enhanced versions to train the enhancement model. The following subsections detail the datasets prepared for training the model.

#### FingerPass Database

The training data were fingerprints from the AES2501 sensor from the FingerPass dataset, which includes 8460 images of different qualities: excellent, very good, good, fair, and poor. To help the model learn how to enhance fingerprints with different quality levels, all fingerprints were enhanced using the HONG method [12], which were used as the target fingerprints.

The proposed method was trained using a minibatch SGD with Adam optimizer considering the following parameters: Momentum parameters *β*1 = 0.5 and *β*2 = 0.999, Learning rate 0.002, μ=100, and *λ* = 0.001.

### 4.2. Testing Details

The performance of the proposed method was tested using two benchmark public databases: FingerPass [3] and MOLF [5].

#### 4.2.1. Multisensor Optical and Latent Fingerprint (MOLF) Dataset

This dataset includes images captured by using three different sensors, having the same sensor technology (optical sensors) and the same capturing method (press). Images in the database come from 100 subjects, where each one of the 10 fingerprints was captured in two sessions (two independent instances were captured in each session). Each sensor was used to capture 4000 images with 1000 fingerprint classes.

Live-scan images in the database are categorized into three subsets. DB1, DB2, and DB3. It can be noted from Figure 2 that those images are visually different due the acquisition sensor used and the capturing process applied.

#### 4.2.2. FingerPass Database

FingerPass consists of images of the same eight fingers (thumb, index finger, middle finger, and ring finger of both hands) captured using nine sensors from 90 subjects; a sample is shown in Figure 1.

It includes two technological types (optical and capacitive sensors) and two capturing methods (in this case, press and sweep). Each subject was asked to take 12 impressions for each finger. Therefore, the database includes images of 720 fingers, where the total number of impressions for one sensor is 90 × 8 × 12 = 8640 images.

Since our model is trained on fingerprints of size 300 × 300 × 1, the fingerprints from the MOLF dataset and FingerPass are preprocessed to match the required size.

## 5. Experimental Results

In this section, we introduce the metric used to evaluate our results and present the outcome of the conducted experiments.

### 5.1. Fingerprint Image Quality Analysis

The NFIQ module of NBIS proposed in [6] was used to analyze the ability of the proposed enhancement algorithm to enhance the quality of cross-sensor fingerprints. The analysis offers a value between 1 and 5, where 1 represents the best quality while 5 represents the worst quality. The score distribution before and after applying the enhancement method was assessed using fingerprints from MOLF and FingerPass datasets to evaluate the performance. The results for MOLF enhancement using adversarial learning are shown in Table 4.

It can be noticed from Table 4 that all images were enhanced, although different sensors were used to capture them. In DB1, there is a significant image quality enhancement, where 3796 images were enhanced out of 4000 to be in class excellent. The difference here is 204 images, which are enhanced compared to the original images.

Moreover, DB2 shows enhancement in class excellent results from 1340 to 2255 and a noticed reduction in a class fair and poor with 27 and 89 images before and 8 and 4 images after for each class. DB3 shows an increase in class excellent fingerprints by 1285 images and a reduction for all other classes; the number of fingerprints of class poor reduces from 97 to 18 after enhancement.

Two learning methods were applied: namely, conventional learning and adversarial learning. A single network was constructed with a loss function that aims to minimize the distance between the predicted and ground truth to test the impact of conventional learning, as described in Section 3.1. On the other hand, the impact of adversarial learning was tested using two networks: a generator and a discriminator, as described in Section 3.2. The results are shown in Table 5 on MOLF datasets.

It can be noticed from Table 5 that the experiment based on adversarial learning offered better results than the conventional one, although the same network architecture was used to generate the fingerprints.

#### Comparison with the State-of-the-Art Method

There are various studies in the field of fingerprint enhancement, for example, the methods proposed in [7,8,14,15]. Although HONG and CHIK methods are a bit old, their performance is still better than the recent methods for cross-sensor fingerprint enhancement, and, due to this reason, they have been used in recent cross-sensor matching methods [4,22,23,24,25,26]. So, we compared our method with HONG and CHIK methods and a more recent method, i.e., JOSHI method [16].

**Figure 7:**
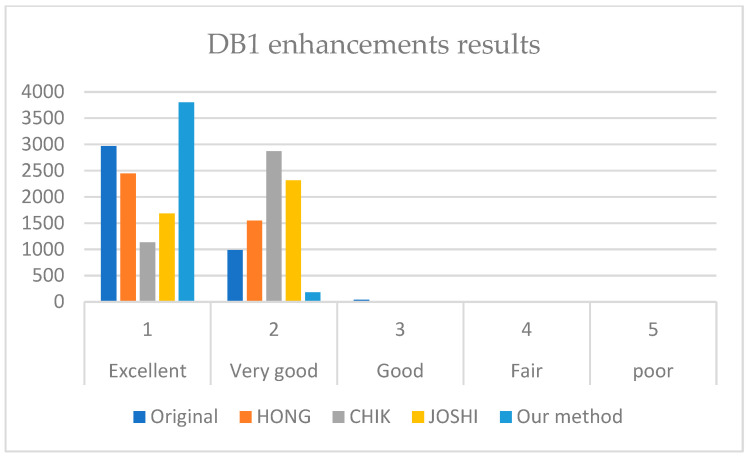
Comparison between the enhancement results of HONG [12], CHIK [13], JOSHI [16], and our method on DB1.

**Figure 8:**
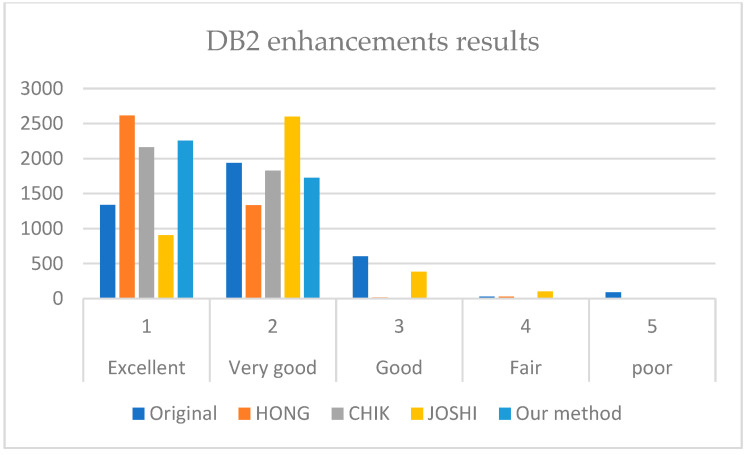
Comparison between the enhancement results of HONG [12], CHIK [13], JOSHI [16], and our method on DB2.

**Figure 9:**
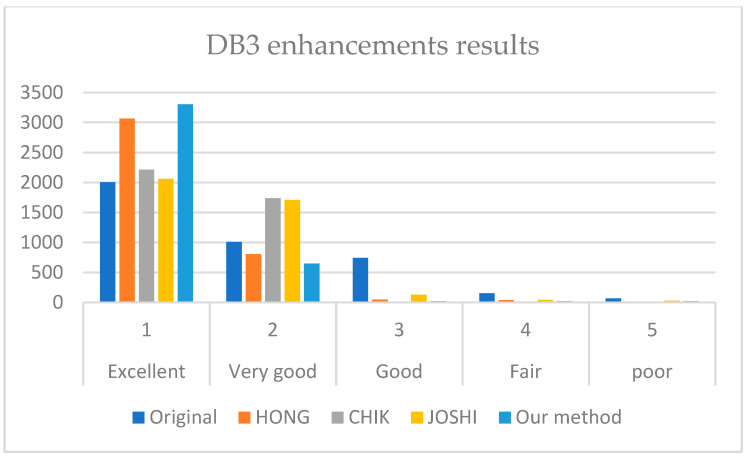
Comparison between the enhancement results of HONG [12], CHIK [13], JOSHI [16], and our method on DB3.

Figure 7, Figure 8 and Figure 9 illustrate the comparison results on DB1, DB2, and DB3.

It is revealed from Figure 7, Figure 8 and Figure 9 that our method outperforms HONG and CHIK methods in enhancing fingerprints to class excellent from DB1 and DB3. For DB2, the number of enhanced fingerprints to class excellent by HONG method is slightly higher than that by our method and CHIK.

### 5.2. Fingerprint Quality Enhancement Index (FQEI)

The FQEI metric was measured using MOLF datasets DB1, DB2, and DB3 by comparing three methods: HONG, CHIK, JOSHI [16], and our method, where obtained results are provided in Table 6. It can be clearly noticed that our method outperformed both HONG, CHIK, and JOSHI methods on DB1, DB2, and DB3.

For DB1, the HONG method performance is 0.2581 since the $E_{s}$ is 348, which is less than the $D_{s}$ (−808). This means that the number of images above the diagonal is less than the images below the diagonal. The same case is for CHIK performance, where the $E_{s}$ is 168, while the $D_{s}$ is −1943 since a large number of fingerprints was degraded from excellent class to very good class. In contrast, our method has a higher enhancement score than the degradation score. Thus, our method outperformed both the HONG and CHIK methods on DB1, DB2, and DB3.

Table 7, Table 8, Table 9, Table 10 and Table 11 illustrate a comparison between the enhancement results obtained with HONG method, CHIK method, JOSHI method, and our method for FingerPass datasets using NFIQ and our metric FQEI.

From Table 7 for FingerPass dataset before enhancement, it can be noticed that there are three sensors that have the highest number of images in poor class, including AES3400, ATRUA, and FPC sensors with 1398, 3107, and 2507 images, respectively.

Based on comparing the results of NFIQ for the three methods after enhancement, it can be noticed that our method offered the highest enhancement in these three sensors by extremely reducing it to zero poor images for the first sensor, one poor image for the second sensor and zero poor images for the third sensor. Moreover, it particularly enhanced the number of images in excellent class to more than 8000 images for the first sensors and the URU4000B sensor. In contrast, the HONG method revealed the highest enhancement for AES2501 sensor. There are also two sensors with the highest number of images in the excellent class: the WS and V300 sensors.

The overall results show that our method outperformed mostly in increasing the number of images in the excellent class. The CHIK method usually transforms fingerprints’ quality to excellent and very good classes but with a noticeable reduction in the number of images in excellent class in most sensors. JOSHI method increases the number of poor fingerprints in two sensors: AES3400 and V3000.

In terms of FQEI metric, our method shows the highest results for five out of nine sensors. The results on AES3400, ATRUA, and URU4000B sensors are 0.9149, 0.9388, 0.9707, respectively, which are very close to 1, and hence a very high enhancement performance. However, a negative enhancement was achieved by JOSHI method in two sensors: AES3400 and V3000. On the other hand, CHIK method gave FQEI of −0.0012 for AES3400 sensor, where the minus sign means distortion in images, which can be obviously noticed by comparing it with the confusion matrix results as shown in Table 12, where most images preserved in good class without enhancement as well as a slight enhancement was revealed from poor class to good class.

### 5.3. Structural Similarity Index Metric (SSIM)

Fingerprint enhancement algorithms are applied to improve fingerprints without changing the ridge structure. This feature can be assessed by computing the SSIM [21] on the generated fingerprints using anguli and their related ground truth, due to the lack of databases that include low-quality images and relative high-quality images. In other words, the higher the obtained SSIM value is, the higher the preserved structural similarity between the generated and ground truth is. Moreover, this denotes that the ridge structure is also maintained.

A comparison was conducted for fingerprints that were enhanced using HONG method, CHIK method, and our method. The test datasets contain two thousand synthetic fingerprints generated using anguli [27]. It is an open-source implementation from the fingerprint generator SFinGe [28] based on simulating synthetic live fingerprints having similar features, such as real-live fingerprints. Two thousand (2000) synthetic fingerprint images produced by Anguli are used to test the model with pattern types following the normal distribution, including the arch, right loop, left loop, whorl, and double loop. From those images generated using Anguli, other input images with lower quality were generated by adding Gaussian noise with morphological operations and blurring the filtering in the frequency domain.

Both the mean and standard deviation of SSIM were then computed as shown in Table 13.

The mean of SSIM between the enhanced fingerprint generated using our model and the ground truth is 0.5127. It can be noticed that our method had the highest mean of SSIM, which means that the preservation of ridge patterns is the best in our method.

### 5.4. Computation Time

The average computation time needed to enhance the URU4000b sensor dataset was computed. All three methods were applied on the same environment (R2021b). The experiment was also applied using a laptop with an Intel Core i7-9750H CPU at 2.60 GHz -2.59 GHz, 32.0 GB RAM, Microsoft Windows 10 in the 64-bit operating system, and an x64-based processor. Our method is faster than HONG, CHIK, and JOSHI [16] methods as shown in Table 14.

## 6. Discussion

The fingerprint sensor interoperability focuses on addressing how the fingerprint-matching system is able to compensate for the differences in the captured fingerprints for the same person by several sensors. The main causes of such variability in fingerprints are the differences in the used capturing technology of sensors, scanning area, sensor resolution, and interaction type.

In practice, each sensor generates its specific type of distortions. Hence, there is a need to enhance captured fingerprints by various sensors. To achieve this, a cross-sensor enhancement method was designed and trained using fingerprints from one sensor, which is the AES2501. On the other hand, this method revealed general enhancement results for other sensors in FingerPass and MOLF datasets. The learning approach considered is the adversarial learning one, which offers better enhancement than the conventional learning one. Moreover, it was found that there was no change in the global flow of ridge patterns within the captured fingerprints by different sensors. This proves its robustness to discrimination. Hence, the edge loss, *L*1 loss, and adversarial loss function were used as loss functions.

The use of dilation convolution offered better enhancement results than those measured using convolution only. This means that the small fingerprint details, considered important features for determining the identity, such as the minutia point and edges, were preserved. This is clearly illustrated in Table 15.

Based on comparing the results of our method with those of two state-of-art fingerprint methods: HONG and CHIK and a more recent method i.e., JOSHI method [16], using two metrics, our method outperformed both of them. However, the NFIQ metric does not offer a precise description for enhancement performance. Therefore, a new metric was designed, called FQEI. This metric gives one result value between 1 and −1 instead of the five classes results as in the NFIQ.

**Figure 10:**
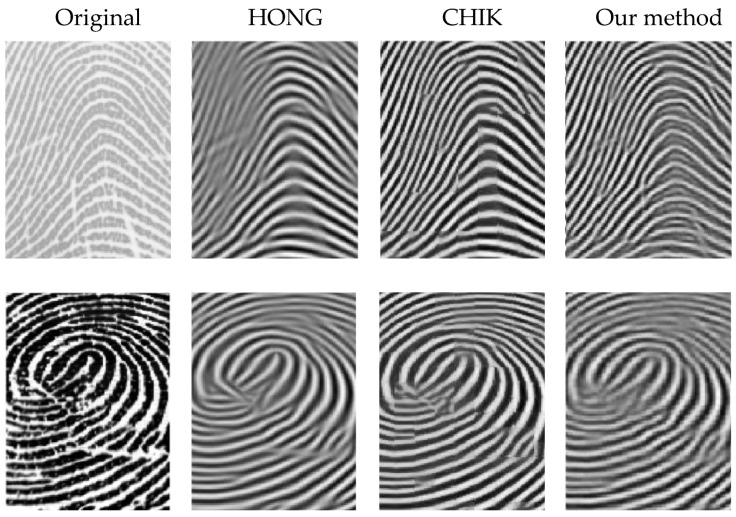
A zoomed-in view for fingerprint enhancement result, where the first column shows the original fingerprint, while the second, third, and fourth columns show those of the HONG [12], CHIK [13] and our method, respectively.

Figure 10 illustrates zoomed-in views of the fingerprints enhanced using the three methods. From the enhanced fingerprints examples shown in Figure 10, it can be noticed that the smoothed ridges related to the processed fingerprints by the HONG method were more enhanced than those of the CHIK method. On the other hand, our method enhanced fingerprints with preserving their original ridge pattern better than HONG and CHIK.

From Table 12, it is obvious that our method offers faster enhancement results than those of HONG, CHIK, and JOSHI methods. In other words, the average computation time needed to enhance one fingerprint by the HONG, CHIK, JOSHI, and our method was 0.63, 0.48, 0.38, and 0.087 s, respectively. Thus, our method is 13% faster than HONG method. However, there are two sensors FX3000 and V300 with less results than what was expected since the fingerprint nature is different than the original data.

## 7. Conclusions

It can be concluded that with the continuous developments in both fingerprint sensor technologies and the Internet of Things (IoT), the use of biometric fingerprint identification has been increasing over the years. Differences in sensor technologies and resolution can lead to different types of distortion, which affects fingerprint image quality. Therefore, fingerprints must be enhanced. On the other hand, there are no sufficient investigations of the cross-sensor enhancement problem in the related literature. Therefore, this paper proposed an efficient solution for this problem based on deep learning, in which cGAN framework is used for training the image-to-image transformation for fingerprint enhancement. It was demonstrated that the proposed method significantly enhanced the cross-sensor fingerprints regardless of the sensor type used. However, there is still space to achieve more enhancement. One of the suggested future works is to explore different loss functions to preserve and recover the ridge patterns.

## Figures and Tables

**Table 1:** Specifications of encoder and decoder models; FS represents the filter size; FN is number of filters and S represents the stride.

Encoder				Decoder			
Layer	FS	FN	S	Layer	FS	FN	S
Conv1_1	3	64	1	Conv1_1	3	64	1
Conv1_2	3	64	1	Conv1_2	3	64	1
Max Pooling 1	2	-	2	Max Un pooling 1	2	-	2
Dilated Conv 2_1	3	64	1	Conv 2_1	3	64	1
Dilated Conv 2_2	3	64	1	Conv 2_2	3	64	1
Max Pooling 2	2	-	2	Max Un pooling 2	2	-	2
Dilated Conv 3_1	3	64	1	Conv 3_1	3	64	1
Dilated Conv 3_2	3	64	1	Conv 3_2	3	64	1
Max Pooling 3	2	-	2	Max Un pooling 3	2	-	2
Dilated Conv 4_1	3	64	1	Conv 4_1	3	64	1
Dilated Conv 4_2	3	64	1	Conv 4_2	3	64	1
Max Pooling 4	2	-	2	Max Un pooling 4	2	-	2
Dilated Conv 5_1	3	64	1	Conv 5_1	3	64	1
Dilated Conv 5_2	3	64	1	Conv 5_2	3	64	1
Max Pooling 5	2	-	2	Max Un pooling 5	2	-	2
				Conv 6_1	3	1	1
				Tanh	-	-	-

**Table 2:** The quality confusion matrix (QCM).

*Q*_11_	*Q*_12_	*Q*_13_	*Q*_14_	*Q*_15_
*Q*_21_	*Q*_22_	*Q*_23_	*Q*_24_	*Q*_25_
*Q*_31_	*Q*_32_	*Q*_33_	*Q*_34_	*Q*_35_
*Q*_41_	*Q*_42_	*Q*_43_	*Q*_44_	*Q*_45_
*Q*_51_	*Q*_52_	*Q*_53_	*Q*_54_	*Q*_55_

**Table 3:** The weight quality matrix (WQM).

0	1	2	3	4
−1	0	1	2	3
−2	−1	0	1	2
−3	−2	−1	0	1
−4	−3	−2	−1	0

**Table 4:** NFIQ quality scores on the Original MOLF dataset and the enhanced dataset by our model (After E.). The up arrow represents better enhancement.

Quality	*Q*	DB1		DB2		DB3	
		Original	After E.	Original	After E.	Original	After E.
Excellent	1	2965	3796 ↑	1340	2255 ↑	2018	3303 ↑
Very good	2	985	183	1940	1724	985	646
Good	3	37	2	603	8	744	16
Fair	4	12	19	27	8	155	19
poor	5	0	0	89	5	97	18

**Table 5:** The effect of the learning approach on the quality of the MOLF database.

**Quality Score**	***Q***	**DB1** **Original**	**Conventional Learning** **(One Net)**	**Adversarial Learning** **(Two Net)**
Excellent	1	2965	3827	3796
Very good	2	985	123	183
Good	3	37	12	2
Fair	4	12	36	19
poor	5	0	2	0
**Quality Score**	***Q***	**DB2** **Original**	**Conventional Learning** **(One Net)**	**Adversarial Learning** **(Two Net)**
Excellent	1	1340	1915	2255
Very good	2	1940	2057	1724
Good	3	603	18	8
Fair	4	27	6	8
poor	5	89	4	5
**Quality Score**	***Q***	**DB3** **Original**	**Conventional Learning** **(One Net)**	**Adversarial Learning** **(Two Net)**
Excellent	1	2018	3206	3303
Very good	2	985	634	646
Good	3	744	39	16
Fair	4	155	86	19
poor	5	97	35	18

**Table 6:** FQEI values computed for HONG method, CHIK method, JOSHI [16] method, and our method for the MOLF dataset.

The Enhancement Method	DB1	DB2	DB3
HONG [12]	0.2581	0.6342	0.7026
CHIK [13]	0.0231	0.5562	0.6508
JOSHI [16]	0.2012	0.1723	0.3270
Our method	0.8863	0.6760	0.8740

**Table 7:** Analysis of the fingerprint quality scores measured by NFIQ of the FingerPass database before enhancement.

Quality	*Q*	AES2501	AES3400	ATRUA	FPC	FX3000	UPEK	V300	WS	URU4000B
Excellent	1	5519	0	28	40	4105	2016	7917	7395	4697
Very Good	2	2423	65	3149	508	4195	5472	637	895	3263
Good	3	662	7177	2356	5585	330	1142	76	304	647
Fair	4	32	0	0	0	0	0	10	42	33
Poor	5	4	1398	3107	2507	10	10	0	4	0

**Table 8:** Analysis of the fingerprint quality scores measured by NFIQ and FQEI of the FingerPass enhanced using HONG method [12].

NFIQ	*Q*	AES2501	AES3400	ATRUA	FPC	FX3000	UPEK	V300	WS	URU4000B
Excellent	1	6194	0	1136	13	4758	945	6381	6786	5245
Very Good	2	2443	202	6852	6020	3882	7693	2258	1853	3389
Good	3	2	8161	546	2596	0	2	1	0	0
Fair	4	1	0	1	0	0	0	0	1	0
Poor	5	0	277	105	10	0	0	0	0	6
**FQEI**		0.6146	0.1110	0.5868	0.4791	0.4497	0.1379	0.5065	0.5829	0.5912

**Table 9:** Analysis of the fingerprint quality scores measured by NFIQ and FQEI of the FingerPass enhanced using CHIK method [13].

NFIQ	*Q*	AES2501	AES3400	ATRUA	FPC	FX3000	UPEK	V300	WS	URU4000B
Excellent	1	4834	0	318	4	2253	838	3953	6217	545
Very Good	2	3806	124	7525	5743	6387	7800	4687	2423	8095
Good	3	0	7323	706	2848	0	2	0	0	0
Fair	4	0	0	0	0	0	0	0	0	0
Poor	5	0	1193	91	45	0	0	0	0	0
**FQEI**		0.5335	−0.0012	0.5410	0.4615	0.2562	0.1372	0.3202	0.5373	0.0683

**Table 10:** Analysis of the fingerprint quality scores measured by NFIQ and FQEI of the FingerPass enhanced using JOSHI method [16].

NFIQ	*Q*	AES2501	AES3400	ATRUA	FPC	FX3000	UPEK	V300	WS	URU4000B
Excellent	1	2216	197	4583	783	2418	2607	2874	2506	2160
Very Good	2	6497	0	3975	6394	6005	5672	1648	6126	6474
Good	3	27	3459	28	1453	189	359	2438	6	6
Fair	4	0	1877	3	8	22	1	464	2	0
Poor	5	0	3107	51	2	6	1	1216	0	0
**FQEI**		0.2291	−0.3792	0.7889	0.5660	0.1027	0.3114	−0.070	0.3096	0.1582

**Table 11:** Analysis of the fingerprint quality scores measured by NFIQ and FQEI of the FingerPass enhanced using our method.

NFIQ	*Q*	AES2501	AES3400	ATRUA	FPC	FX3000	UPEK	V300	WS	URU4000B
Excellent	1	5824	8192	8066	3958	4700	2924	4743	6779	8134
Very Good	2	2797	65	562	4680	2609	5716	241	1855	467
Good	3	2	82	6	1	813	0	3343	2	23
Fair	4	17	301	5	1	399	0	206	4	16
Poor	5	0	0	1	0	119	0	107	0	0
**FQEI**		0.5645	0.9388	0.9707	0.7836	0.3149	0.3407	0.2931	0.5825	0.9149

**Table 12:** Quality Confusion Matrices for AES3400 sensor enhancements using: (**a**) Hong [12] (**b**) CHIK [13] (**c**) Our method.

	(a)					(b)					(c)				
	*Q*1	*Q*2	*Q*3	*Q*4	*Q*5	*Q*1	*Q*2	*Q*3	*Q*4	*Q*5	*Q*1	*Q*2	*Q*3	*Q*4	*Q*5
q1	0	0	0	0	0	0	0	0	0	0	0	61	6821	0	1310
q2	0	17	144	0	41	0	15	99	0	10	0	1	48	0	16
q3	0	47	6847	0	1267	0	46	6450	0	827	0	1	66	0	15
q4	0	0	0	0	0	0	0	0	0	0	0	2	242	0	57
q5	0	1	186	0	90	0	4	628	0	561	0	0	0	0	0

**Table 13:** Mean and standard deviation (std) of SSIM.

The Enhancement Method	Mean of SSIM	Std
HONG [12]	0.4551	0.0482
CHIK [13]	0.4650	0.0460
JOSHI [16]	0.4125	0.0354
Our method	0.5127	0.0693

**Table 14:** Comparison between the computation time for enhancement.

Method	Average Computation Time (in Seconds)
HONG [12]	0.63
CHIK [13]	0.48
JOSHI [16]	0.38
Our method	0.087

**Table 15:** The impact of using dilation operation and convolution operation for MOLF datasets.

FQEI	DB1	DB2	DB3
Convolution layer	0.8643	0.5208	0.7996
Dilation Convolution	0.8863	0.6760	0.8740

## Data Availability

The MOLF dataset is available at: http://research.iiitd.edu.in/groups/iab/molf.html, and the FingerPass (accessed on 22 May 2021) dataset is available at: http://www.fingerpass.csdb.cn/ (accessed on 22 May 2021).

## Appendix A

To clarify FQEI metric, the following example is provided: For a small dataset of 40 fingerprint images having different qualities, the table below represents the NFIQ degrees before (original) and after the enhancement. The QCM is then computed, where the first column represents the total number of images before enhancement, while the first row represents the total number of images after enhancement and so on. Both the IS and WS are then computed as follows:

IS=10×1+10×2+5×3+10×4WS=5×−4+10×−3+10×−2+5×−1

**Table A1:** NFIQ quality score of the example before and after enhancements.

NFIQ Values	*Q*1	*Q*2	*Q*3	*Q*4	*Q*5
Original	5	10	10	5	10
The enhanced	20	4	14	1	1

**Table A2:** Computing the (QCM).

	*Q*1	*Q*2	*Q*3	*Q*4	*Q*5
q1	1	8	5	2	4
q2	0	1	2	0	1
q3	3	0	3	3	5
q4	1	0	0	0	0
q5	0	1	0	0	0

**Table A3:** Computing the WCM by Multiplying QCM with WQM.

	*Q*1	*Q*2	*Q*3	*Q*4	*Q*5
q1	0	8	10	6	16
q2	0	0	2	0	3
q3	−6	0	0	3	10
q4	−4	0	0	0	0
q5	0	−3	0	0	0

**Table A4:** Calculating the FQEI.

$E_{s}$	$D_{s}$	IS	WS	ER	DR	FQEI
58	−13	85	−75	0.6823	−0.1733	0.509
